# Isolated Mediastinal Myeloid Sarcoma after *NPM1*-Positive Pediatric Acute Myeloid Leukemia

**DOI:** 10.4274/tjh.galenos.2019.2018.0434

**Published:** 2019-11-18

**Authors:** Özlem Tüfekçi, Şebnem Yılmaz, Melek Erdem, Birsen Baysal, Hale Ören

**Affiliations:** 1 Dokuz Eylül University Faculty of Medicine, Department of Pediatric Hematology, İzmir, Turkey

**Keywords:** Acute myeloid leukemia, sMyeloid sarcoma, Mediastinal mass, NPM1

## To the Editor,

Myeloid sarcoma (MS) is a rare extramedullary mass that consists of immature myeloid cells. The most common locations are the soft tissue, bone, periosteum, orbit, and lymph nodes [1,2]. Mediastinal involvement is very rare and most commonly reported with concurrent bone marrow involvement [3]. Herein we present a previously treated nucleophosmin (*NPM1*)-positive acute myeloid leukemia (AML) patient who later presented with isolated mediastinal MS.

**Figure 1:**
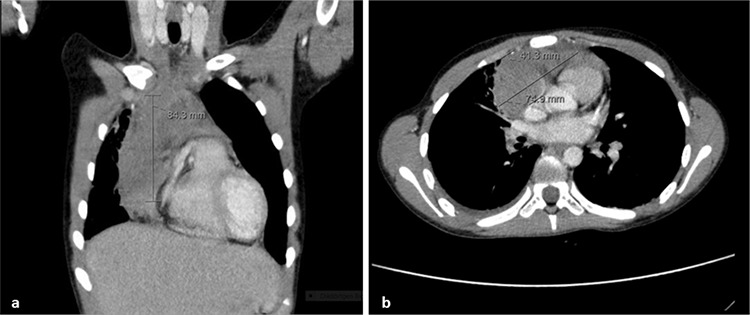
Computed tomography of the thorax showing anterior mediastinal mass in coronal (a) and axial (b) sections.

A 9-year-old female patient presented with fatigue and weakness. Physical examination revealed no pathological findings. Blood tests demonstrated hemoglobin of 12.2 g/dL, hyperleukocytosis (100,500/µL), and thrombocytopenia (43,000/µL) with 88% blasts in the peripheral blood smear. Bone marrow aspirate revealed 90% blasts with M1 subtype. Treatment was started according to the AML-BFM 2012 protocol. Conventional cytogenetic analysis failed due to lack of spontaneous mitosis and fluorescent in situ (FISH) analysis for t(8;21), inv(16), t(15;17), and t(9,22) from bone marrow samples revealed negative results. Molecular genetic analysis in the peripheral blood showed *NPM1 *positivity and *FLT3-ITD* negativity. Morphologic and molecular remission was obtained at the end of the first induction block. She presented with back pain and fever seven months after cessation of maintenance treatment. Computed tomography (CT) of the thorax showed a solid mass of 84x75x41 mm in the anterior mediastinum (Figure 1). Bone marrow examination was normal; however, peripheral blood showed *NPM1* positivity. Conventional cytogenetic analysis from the bone marrow was within normal limits, while *NPM1* could not be studied from bone marrow. Her previous CT scans that were performed for investigation of invasive pulmonary aspergillosis were all normal. Fine-needle aspiration biopsy of the mass was performed; histopathological examination revealed myeloblasts that were positive for myeloperoxidase, CD15, and CD33. Microscopic examination of the imprint of the biopsy also revealed myeloblasts of M1 subtype (Wright stain). Major reduction in tumor mass (7 mm residual tumor) and *NPM1* negativity were achieved after one block of FLAG (fludarabine, cytarabine, filgrastim) and two blocks of FLAG-mitoxantrone. The patient underwent successful bone marrow transplantation from a matched unrelated donor and has been in remission for one year.

MS of the mediastinum is very rare; most of the cases have been reported as initial presentation with concurrent bone marrow involvement [3,4,5]. MS as a relapse has been more frequently reported in post-transplant patients compared to those treated without allogeneic hematopoietic stem cell transplantation [6,7]. Our patient is unique as she presented with isolated mediastinal MS after chemotherapy treatment. Another important point about our patient is that the *NPM1* positivity was detected at the same time as MS. The incidence of MS has been known to be higher in certain cytogenetic abnormalities, in particular t(8,21) [1,6]. Falini et al. [8], in their study with 181 MS samples, identified *NPM1* mutations as the most frequent molecular lesion in MS, defining the molecular status in 15% of cases. Our patient was negative for t(8:21) but had *NPM1 *positivity.

In conclusion, even though *NPM1* is not a poor prognostic factor for AML, it should be kept in mind that patients with *NPM1* positivity may later present with MS, as in the case of our patient, who presented with isolated MS of the mediastinum months after cessation of chemotherapy.
